# Identification of Two Novel Candidate Genetic Variants Associated With the Responsiveness to Influenza Vaccination

**DOI:** 10.3389/fimmu.2021.664024

**Published:** 2021-07-01

**Authors:** Simin Wen, Hejiang Wei, Qijun Liao, Mao Li, Shuyi Zhong, Yanhui Cheng, Weijuan Huang, Dayan Wang, Yuelong Shu

**Affiliations:** ^1^ School of Public Health (Shenzhen), Sun Yat-sen University, Guangzhou, China; ^2^ National Institute for Viral Disease Control and Prevention, Chinese Center for Disease Prevention and Control, Beijing, China

**Keywords:** influenza, vaccine, immune response, genetic variant, genome-wide association study, ZBTB46, IQGAP2

## Abstract

**Background:**

Annual vaccination is the most effective prevention of influenza infection. Up to now, a series of studies have demonstrated the role of genetic variants in regulating the antibody response to influenza vaccine. However, among the Chinese population, the relationship between genetic factors and the responsiveness to influenza vaccination has not been clarified through genome-wide association study (GWAS).

**Method:**

A total of 1,968 healthy volunteers of Chinese descent were recruited and 1,582 of them were available for the subsequent two-stage analysis. In the discovery stage, according to our inclusion criteria, 123 of 1,582 subjects were selected as group 1 and received whole-genome sequencing to identify potential variants and genes. In the verification stage, 29 candidate variants identified by GWAS were selected for further validation in 481 subjects in group 2. Besides, we also analyzed nine variants from previously published reports in our study.

**Results:**

Multivariate logistic regression analysis showed that compared with the TT genotype of *ZBTB46* rs2281929, the TC + CC genotype was associated with a lower risk of low responsiveness to influenza vaccination adjusted for gender and age (Group 2: *P* = 7.75E-05, OR = 0.466, 95%CI = 0.319–0.680; Combined group: *P* = 1.18E-06, OR = 0.423, 95%CI = 0.299–0.599). In the combined group, *IQGAP2* rs2455230 GC + CC genotype was correlated with a lower risk of low responsiveness to influenza vaccination compared with the GG genotype (*P* = 8.90E-04, OR = 0.535, 95%CI = 0.370–0.774), but the difference was not statistically significant in group 2 (*P* = 0.008). The antibody fold rises of subjects with *ZBTB46* rs2281929 TT genotype against H1N1, H3N2,and B were all significantly lower than that of subjects with TC + CC genotype (*P* < 0.001). Compared with *IQGAP2* rs2455230 GC + CC carriers, GG carriers had lower antibody fold rises to H1N1 (*P* = 0.001) and B (*P* = 0.032). The GG genotype of rs2455230 tended to be correlated with lower antibody fold rises (*P* = 0.096) against H3N2, but the difference was not statistically significant. No correlation was found between nine SNPs from previously published reports and the serological response to influenza vaccine in our study.

**Conclusion:**

Our study identified two novel candidate missense variants, *ZBTB46* rs2281929 and *IQGAP2* rs2455230, were associated with the immune response to influenza vaccination among the Chinese population. Identifying these variants will provide more evidence for future research and improve the individualized influenza vaccination program.

## Introduction

Influenza is an acute infectious respiratory disease with an annual occurrence of one billion cases worldwide, including three to five million serious cases and 290,000–650,000 deaths (1). For some vulnerable populations such as infants, pregnant women, and elderly people, influenza poses a serious threat to their lives and health. Annual vaccination is the most effective prevention of influenza infection. However, according to the data from the World Health Organization (WHO) (2), the average effectiveness of seasonal influenza vaccine over the last 16 years was only 39.9%, suggesting that current vaccines cannot provide sufficient protection. There are at least two factors that may influence vaccine effectiveness (VE). One is the matching degree of circulating strains and vaccine strains, and the other is the immunogenicity of vaccine. Currently, although a well-developed global influenza surveillance network has been established, the prediction of annual vaccine strains remains a huge challenge. Therefore, it is of great significance to improve the immunogenicity of vaccines in case the vaccine strains do not match circulating strains. Many factors may affect the immune response to influenza vaccination, including gender, age, adjuvant use, delivery mode, and so on. For individuals who share the same features mentioned above, their immune responses to the same vaccine may also vary significantly. This indicates that genetic factors may play a role in the immunologic processes.

Single nucleotide polymorphism (SNP) is one of the most common types of heritable variation in humans. Currently, some studies have observed some SNPs that may affect the responsiveness to influenza vaccination. HLA is a polygenic and polymorphic complex involved in antigen presentation. The relationship between SNPs in HLA and the immune responses to influenza vaccination has been demonstrated (3–7). Besides, some studies also reported that genetic variants in some immune-related genes may also influence the immunological responses (6, 8).

To our knowledge, a genome-wide association study (GWAS) has not yet been performed to examine the genetic factors that were related to the responsiveness to influenza vaccination in the population of Chinese descent. Therefore, we conducted a GWAS in healthy volunteers of Han Chinese. A replication study was also conducted to validate the results of the GWAS. In addition, in our cohort, we also validated some SNPs previously reported to be associated with the immune responses to influenza vaccine. Identification of these genetic variants may provide more evidence for future research and improve the individualized influenza vaccination program.

## Materials and Methods

### Subjects and Study Design

A total of 1,968 healthy volunteers from September 2009 to September 2019 were enrolled from Yunnan Center for Disease Control and Prevention and Urumqi Center for Disease Control and Prevention. All participants were administered intramuscular injections of trivalent inactivated vaccine (TIV) which contains 15 μg hemagglutination (HA) before the flu season. The vaccine strains of TIVs used in our study were consistent with the northern hemisphere vaccine component recommended by WHO. Blood samples were collected before and 28 days after the vaccination. The exclusion criteria were as follows: not Han Chinese, lost to follow-up, inadequate blood samples, and repeated vaccination. In the end, 386 subjects were excluded, and 1,582 subjects in total were suitable for further research.

According to the age of subjects, we divided them into four groups: infants (≤5 years), children (6–17 years), adults (18–64 years) and elderly people (≥65 years). Seroprotection rate was defined as a percentage of subjects with post-vaccination titer ≥1:40. Seroconversion rate was defined as a percentage of subjects with either a pre-vaccination hemagglutination inhibition (HAI) titer <1:10 and post-vaccination HAI titer ≥1:40 or a pre-vaccination HAI titer ≥1:10, and a minimum four-fold increase in post-vaccination HAI titer. Considering the results of the HAI test, we selected some of the subjects according to our inclusion criteria and divided them into two groups. In group 1, low responders (LRs) were subjects from phenotypic extremes whose HAI titer reached neither the seroprotection rate nor the seroconversion rate to all three vaccine strains. Under this strict standard, only 41 of 1,582 subjects were eligible to be classified as LRs in group 1. According to the age and gender of these 41 LRs, 82 matched subjects were selected as responders from the cohort, the matching ratio was 1:2. Responders were defined as those whose HAI titers reached both the seroprotection rate and the seroconversion rate to all three vaccine strains. In this way, a total of 123 healthy volunteers with 41 LRs and 82 responders were included in group 1. In group 2, subjects included in group 1 were all removed. To validate the potential variants in larger sample size, we broadened the inclusion criteria appropriately. LRs were defined as HAI titer failed to reach the seroconversion rate to all three vaccine strains. And responders were defined as HAI titer reached the seroconversion rate to all three vaccine strains. Therefore, 481 subjects with 193 LRs and 288 responders were included in group 2. The combined group is a combination of group 1 and group 2. All the individuals in group 1 met the inclusion requirements of group 2.

**Figure 1:**
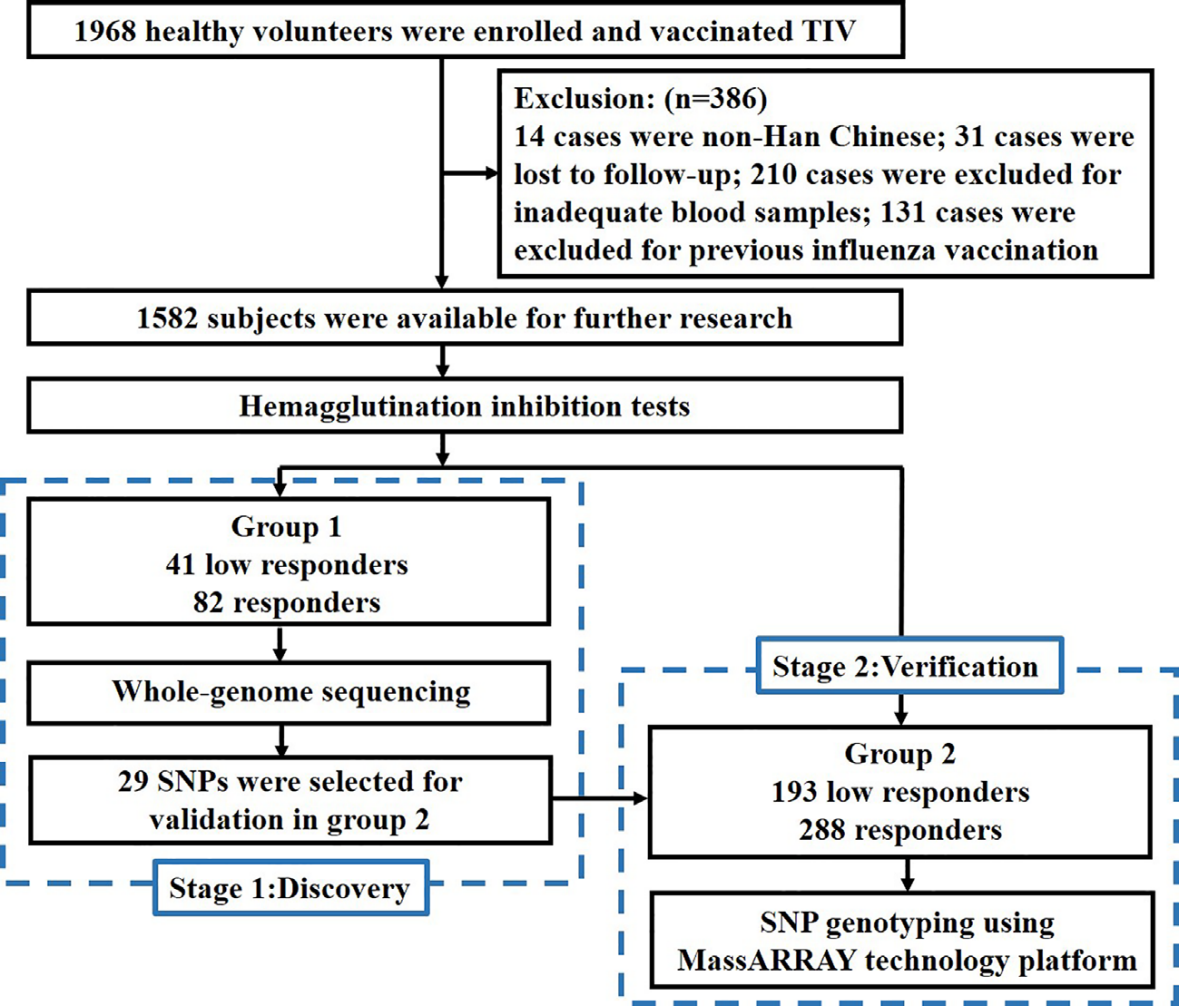
Study flow chart.

In this study, we conducted two-stage sequencing. In the discovery stage, 123 subjects in group 1 received whole-genome sequencing (WGS) to identify potential variants and genes through GWAS and gene-based association study. In the verification stage, 29 potential variants from GWAS were selected for genotyping to validate the results in group 2. The flow chart of this study is shown in **Figure 1**.

### Whole-Genome Sequencing and Quality Control

Genomic DNA was extracted from peripheral blood leukocytes using a TIANamp Blood DNA kit (TIANGEN BIOTECH (Beijing) Co., LTD, China) according to the manufacturer’s instructions. We performed WGS on the BGISEQ-500 sequencing platform with paired-end 100 base run. The average sequencing depth was 39.09×, and the coverage rate was 96.49% (**Supplementary Table 1**). Raw reads were filtered by removing adapters and low-quality sequences; the remaining clean reads of each sample were aligned to hg38 using the Burrows–Wheeler Aligner (BWA) software (version 0.75) (9). Duplicated reads were marked using Picard (https://broadinstitute.github.io/picard). Base quality score recalibration was then performed using the Genome Analysis Toolkit (GATK v3.7) (10). SNPs and small insertions/deletions (indels) were detected by GATK HaplotypeCaller and GenotypeGVCFS tools according to the best practices recommended. To obtain high-quality variants, we applied a series of exclusion criteria to remove variants that had an average depth <8, a minor allele mean depth <4, <90% samples covered at least eight reads, a low mapping quality score, strand bias or allelic imbalance, and deviations from Hardy–Weinberg equilibrium (HWE) (**Supplementary Table 2**). Retained variants were annotated using ANNOVAR (11).

For sample quality control, samples with a mean sequencing depth <30, <90% genome covered by at least 10×, inbreeding coefficient >0.1 or <−0.1, a 2nd-degree or closer relationship with other samples or a population outlier (smartpca) (12) were excluded. Therefore, a total of 12 samples including seven LRs and five responders were removed according to these exclusion criteria.

### SNP Selection and Genotyping

According to the results of GWAS, six highly significant variants (*P <*1 × 10^−5^) were selected for further validation. To identify more potential variants, we also focus on SNPs (*P <*1 × 10^−2^) in coding region. Considering the function of genes, 23 exonic SNPs of immune-related genes were selected. Therefore, a total of 29 variants were included in the subsequent verification analysis. In addition, we also validate nine previously reported SNPs associated with the responsiveness to influenza vaccination. And the minor allele frequencies (MAFs) of these nine SNPs were no less than 0.05 in the Chinese Han population according to the 1000 Genomes Project data. Genotyping of each SNP was conducted using the MassARRAY technology platform (Sequenom, San Diego, California, USA) and determined by BioMiao Biological Technology (Beijing, China). Genotyping was performed by technicians who were blinded to the study design. According to the number of mutations carried by the subjects, their genotypes were classified into wild-type homozygote, mutant heterozygote, and mutant homozygote. SNPs with HWE-*P <*0.001 or call rate <95% were excluded.

### HAI Test

Serological specimens from all subjects were separated and stored at −30°C. Red blood cells (RBCs) of turkey were used for influenza A/H1N1 and B HAI assays, while RBCs of guinea pig were used for influenza A/H3N2 HAI assays. Before the detection, receptor destroying enzyme (RDE) was mixed with the serum in the ratio of 3:1 and placed in a 37°C water bath for 16–18 h to remove the non-specific inhibitor. Then, the mixture was bathed in water at 56°C for 30 min to inactivate the RDE. The HAI titer was designated to be the highest serum dilution to inhibit hemagglutination. HAI assays were performed against the northern hemisphere influenza vaccine strains according to the standardized protocol by Technical Guidelines for National Influenza Surveillance (13). The recommended vaccine strains from 2009 to 2019 were summarized in **Supplementary Table 3**.

### Prediction of SNP Function

The prediction of SNP function was performed using SNPinfo (https://snpinfo.niehs.nih.gov/) provided by the National Institute of Environmental Health Sciences (NIEHS).

### Genetic Models

In this study, a total of four genetic models (additive genetic model, dominant genetic model, recessive genetic model, and overdominant genetic model) were applied in the analysis. The additive genetic model was used to compare the distribution frequencies of the three genotypes. The other three genetic models divided the subjects into two groups by different combinations of the three genotypes. The dominant genetic model compared the distribution frequencies of wild-type homozygotes with other subjects. The recessive genetic model compared the distribution frequencies of mutant homozygotes with other subjects. The overdominant genetic model divided the subjects into homozygotes and heterozygotes to analyze whether the mutation had heterozygous superiority. According to the results of data analysis, only the optimum genetic model with the highest impact was selected and presented.

### Statistical Analysis

#### Discovery Stage

For all the variants identified in WGS, we used Fisher’s exact test to assess the distribution frequencies of alleles and genotypes respectively. For variants with MAF >5%, we applied logistic regression adjusted for the top four principal components and age. Association analysis was performed by the PLINK (14) (v1.07). A gene-based association analysis was conducted for low-frequency variants with MAF <0.05. We defined two variant groups, including: 1. a “Deleterious (Broad)” set defined as non-sense, splice-site, indel frameshift, and missense, which is annotated as deleterious by at least one of the five protein prediction algorithms of likelihood ratio test (LRT) score, Mutation Taster, PolyPhen-2 HumDiv, PolyPhen-2 HumVar, and SIFT. 2. “Deleterious (Strict)” set comprising non-sense, splice-site, indel frameshift, and missense, which is annotated as deleterious by all five protein prediction algorithms. For variants that have different annotations from multiple transcripts of the gene, each variant with the highest impact was presented. We performed Fisher’s exact test and SKAT (15) (EPACTS v3.2.6, https://genome.sph.umich.edu/wiki/EPACTS) to calculate the gene-based collapsing on these two defined variants sets.

#### Verification Stage

The HWE test for assessing the SNP genotype frequency among subjects was conducted. Assessment of pairwise linkage disequilibrium (LD) was performed by the Haploview V.4.2 software. Categorical data were expressed in frequencies (percentages) and compared by χ^2^ test or Fisher’s exact test when appropriate. Associations between variants and responsiveness to influenza vaccination were calculated by multivariate logistic regression models adjusted for age and gender. When the fold rises of the antibodies are calculated, the HAI titers before and after vaccination were transformed to their logarithms. The antibody fold rises with unnormal distribution through Kolmogorov–Smirnov Test were described as the median [interquartile range (IQR)] and compared by Mann–Whitney U Test. The level of statistical significance was *P <*0.05. In the analysis of the relationship between variants and influenza vaccine response, the significance level should be turned to *P <*1.32E-03 (0.05/38 = 1.32E-03) according to Bonferroni correction. Analyses were performed by the IBM SPSS Statistics (version 25.0). Graphs were processed by GraphPad Prism v 6 (GraphPad Software, CA). The statistical power of the association analysis was estimated using the Quanto software (version 1.2.4).

## Results

### Characteristics of Subjects

In group 1, a total of 123 subjects including 41 LRs as cases and 82 matched subjects as controls received WGS. After the quality control, 12 samples were removed. Therefore, 34 cases and 77controls were finally included in the GWAS. In the verification study conducted in group 2, there were 193 cases (LRs) and 288 controls (responders). As shown in **Table 1**, between LRs and responders, no significant differences were observed in gender and age distribution in all three groups (*P* > 0.05).

**Table 1:** Characteristics of subjects in three groups.

Variants	Group 1 (n = 111)			Group 2 (n = 481)			Combined (n = 592)		
	LR (%) (n = 34)	Responder (%) (n = 77)	*P*	LR (%) (n = 193)	Responder (%) (n = 288)	*P*	LR (%) (n = 227)	Responder (%) (n = 365)	*P*
Gender									
Male	13 (32.5)	27 (67.5)	0.748	78 (40.4)	115 (59.6)	0.915	91 (39.1)	142 (60.9)	0.774
Female	21 (29.6)	50 (70.4)		115 (39.9)	173 (60.1)		136 (37.9)	223 (62.1)	
Age									
≤5	9 (32.1)	19 (67.9)	0.952^a^	45 (40.9)	65 (59.1)	0.077	54 (39.1)	84 (60.9)	0.102
6-17	3 (27.3)	8 (72.7)		13 (26.0)	37 (74.0)		16 (26.2)	45 (73.8)	
18-64	13 (28.3)	33 (71.7)		94 (39.7)	143 (60.3)		107 (37.8)	176 (62.2)	
≥65	9 (34.6)	17 (65.4)		41 (48.8)	43 (51.2)		50 (45.5)	60 (54.5)	

a Fisher exact test. LR, low responder.

### GWAS and Gene-Based Association Analysis

**Figure 2:**
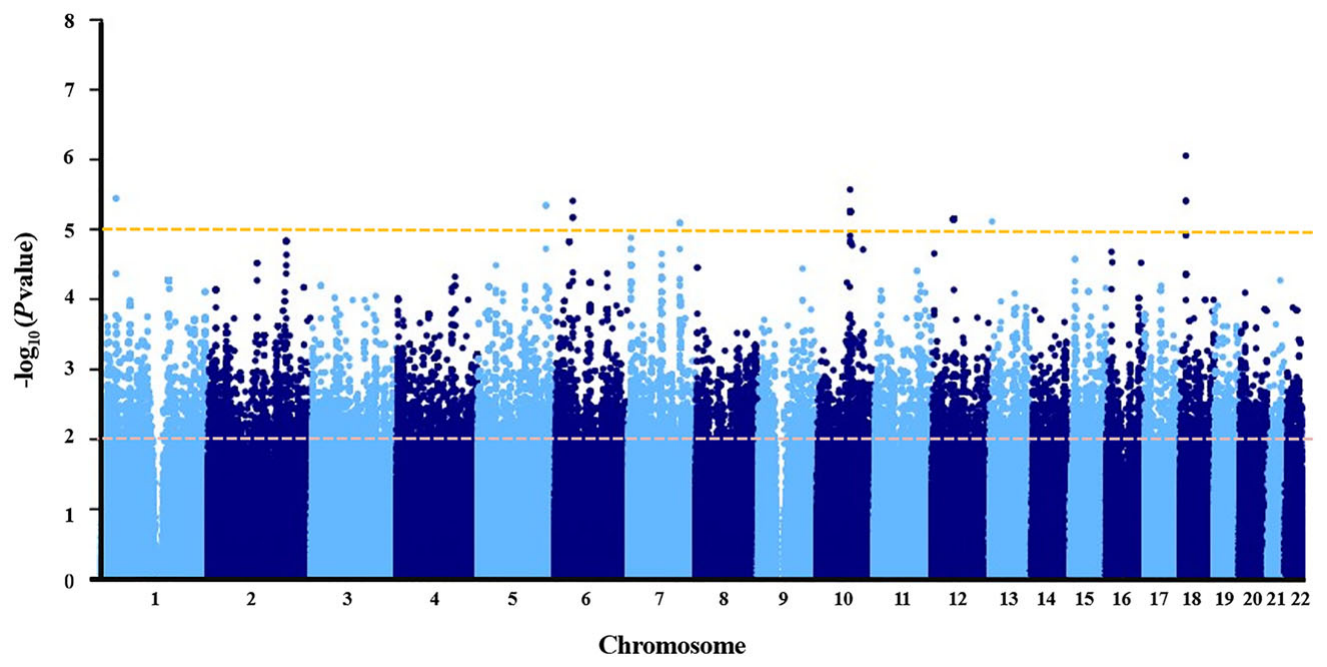
Manhattan plot of negative log of corresponding p‐values from genome‐wide association study.

Association *P* values from GWAS were presented in the Manhattan plot (**Figure 2**). No variant displayed genome-wide significant association (*P* < 1 × 10^−8^). The most significant SNP was *LDLRAD4* rs2847111 (*P* = 8.82E-07) (**Table 2**). A total of 45 highly significant variants (*P* < 1 × 10^−5^) identified by GWAS were summarized in **Supplementary Table 4**, and none of them was in the coding region. Among these variants, only six of them were selected for subsequent analysis according to the function of genes and variants, the location of variants, and the LD between variants (**Table 2**). For variants in LD (r^2^ > 0.8), only one of them was selected. In order to identify more potential variants, we also considered SNPs (*P* < 1 × 10^−2^) in the coding region. Based on previous reports on the function of genes, 23 exonic SNPs of immune-related genes were selected. **Table 2** summarizes the information of 29 SNPs of interest. The 29 associated SNPs indicated a total of 27 potential genes. No studies have reported that these 29 variants are associated with the immune response to influenza vaccine.

**Table 2:** Loci of interest from genome-wide association study.

Number	Chr	Loci	Position	Nearest Gene	Other nearby gene	Minor allele	Major allele	Function	MAF			*P* ^a^	OR (95%CI)
									Case	Control	Database (Chinese)		
1	18	rs2847111	13501672	LDLRAD4		A	G	intronic	0.029	0.305	0.204	8.82E-07	0.069 (0.016-0.294)
2	18	rs3216103	13501276-13501280	LDLRAD4		TAG	DEL	intronic	0.059	0.331	0.277	3.91E-06	0.126 (0.044-0.366)
3	10	rs287187	77423073	KCNMA1		A	T	intronic	0.515	0.201	0.325	5.58E-06	4.208 (2.269-7.803)
4	12	rs1567584	51270520	SMAGP	BIN2	A	C	upstream	0.265	0.045	0.073	7.13E-06	7.560 (2.982-19.160)
5	7	rs2707521	121300382	CPED1	WNT16	T	C	intergenic	0.206	0.526	0.442	8.09E-06	0.234 (0.120-0.456)
6	7	rs2707520	121311090	CPED1	WNT16	A	C	intergenic	0.206	0.526	0.442	8.09E-06	0.234 (0.120-0.456)
7	1	rs11264505	156598460	GPATCH4		G	A	synonymous	0.162	0.013	0.092	5.38E-05	14.670 (3.153-68.210)
8	13	rs1047740	102796852	KDELC1		T	C	missense	0.279	0.546	0.481	2.58E-04	0.323 (0.174-0.599)
9	12	rs879732	12087265	BCL2L14		T	C	synonymous	0.735	0.474	0.495	3.99E-04	3.086 (1.650-5.747)
10	17	rs61644407	601889	VPS53		C	A	missense	0.427	0.195	0.257	4.98E-04	3.074 (1.646-5.739)
11	16	rs75559202	78425018	WWOX		G	C	missense	0.235	0.065	0.107	5.53E-04	4.431 (1.891-10.380)
12	7	rs73683127	18658043	HDAC9		T	G	splicing	0.324	0.123	0.141	6.70E-04	3.398 (1.689-6.837)
13	14	rs5510	94567019	SERPINA4		T	C	synonymous	0.132	0.351	0.325	6.89E-04	0.283 (0.130-0.614)
14	12	rs3210837	51292047	BIN2		T	C	synonymous	0.294	0.104	0.146	6.97E-04	3.594 (1.732-7.494)
15	19	rs306507	55947976	NLRP8		C	T	missense	0.074	0.260	0.170	1.04E-03	0.226 (0.085-0.602)
16	2	rs1044280	8731212	KIDINS220		A	C	missense	0.324	0.143	0.160	3.14E-03	2.870 (1.454-5.662)
17	5	rs1800449	122077513	LOX		T	C	missense	0.324	0.149	0.194	3.89E-03	2.724 (1.388-5.345)
18	14	rs2277484	24160535	RNF31		A	G	missense	0.324	0.149	0.112	3.89E-03	2.724 (1.388-5.345)
19	2	rs16831235	134987559	MAP3K19		A	G	missense	0.235	0.084	0.117	4.16E-03	3.337 (1.503-7.412)
20	7	rs2228410	36724067	AOAH		T	C	missense	0.647	0.435	0.500	5.46E-03	2.381 (1.319-4.297)
21	5	rs2455230	76637140	IQGAP2		C	G	missense	0.368	0.578	0.471	5.46E-03	0.425 (0.236-0.764)
22	18	rs2919643	45839038	SIGLEC15		C	T	missense	0.221	0.416	0.379	6.04E-03	0.398 (0.206-0.768)
23	17	rs17854914	4818081	PLD2		G	A	missense	0.000	0.097	0.049	6.51E-03	—
24	11	rs3816921	12224686	MICAL2		G	C	synonymous	0.235	0.429	0.456	6.59E-03	0.410 (0.215-0.782)
25	6	rs2523855	31054381	HCG22		C	G	missense	0.544	0.344	0.442	7.38E-03	2.274 (1.271-4.069)
26	6	rs2180314	52752933	GSTA2		C	G	missense	0.338	0.169	0.291	7.97E-03	2.516 (1.306-4.848)
27	20	rs2281929	63790727	ZBTB46		C	T	missense	0.338	0.533	0.447	8.72E-03	0.449 (0.248-0.813)
28	16	rs78108426	10907564	CIITA		A	C	missense	0.044	0.175	0.121	9.46E-03	0.217 (0.063-0.743)
29	3	rs3772173	170360444	SKIL		C	T	missense	0.265	0.117	0.194	9.48E-03	2.720 (1.312-5.640)

a Fisher exact test. Chr, chromosome; MAF, minor allele frequency; OR, odds ratio; CI, confidence interval.

For rare variants with low frequency, a gene-based association analysis was conducted. The top 27 genes (*P* < 0.01) identified through Deleterious (Broad) are shown in **Supplementary Table 5**. Two genes including AGBL1 and FAM47E were significant (*P* < 0.01) in the set of Deleterious (Strict). No studies have reported that these genes are associated with the immune response to influenza vaccine.

### Verification Study for 29 Candidate Variants

To verify the findings in GWAS, a validation study was conducted in 193 LRs as cases and 288 responders as controls in group 2. All SNPs tested had a call rate ≥95% and conformed to HWE (*P* > 0.001). The MAF of SNPs in this study was consistent with the published data for the Han Chinese population in the 1000 Genomes Project data, indicating that our data set is reliable (**Supplementary Table 6**). In the univariate association analysis, four SNPs including *SERPINA4* rs5510, *IQGAP2* rs2455230, *HCG22* rs2523855, and *ZBTB46* rs2281929, had different genotype frequencies between the LR group and the responder group (*P* < 0.05). The frequency of *HCG22* rs2523855 C allele (*P* = 0.003) and *ZBTB46* rs2281929 C allele (*P* = 1.75E-04) in the responder group tended to be higher than that in the LR group. However, after the Bonferroni correction (*P* = 1.32E-03), only the distribution frequencies of *ZBTB46* rs2281929 genotypes (*P* = 2.47E-04) and alleles (*P* = 1.75E-04) were still significantly different between the LR group and the responder group (**Supplementary Table 6**). For variants with *P <*0.10 in the univariate analysis, the multivariate logistic regression analyses adjusted for gender and age were conducted. All results were presented using the optimum genetic model (**Table 3**). Multivariate analysis results of all the 29 variants were shown in **Supplementary Table 7**. The results showed that compared with the TT genotype of *ZBTB46* rs2281929, the TC + CC genotype was associated with a lower risk of low responsiveness to influenza vaccination adjusted for gender and age (Group 2: *P* = 7.75E-05, OR = 0.466, 95%CI=0.319–0.680; Combined group: *P* = 1.18E-06, OR = 0.423, 95%CI = 0.299–0.599). In the combined group, *IQGAP2* rs2455230 GC + CC genotype was also correlated with a lower risk of low responsiveness to influenza vaccination compared with the GG genotype (*P* = 8.90E-04, OR = 0.535, 95%CI = 0.370–0.774), but the difference was not statistically significant in group 2 (*P* = 0.008).

**Table 3:** Multivariate logistic regression analysis adjusted for age and gender.

Gene	SNPs	Genetic Model	Genotype		Group 2		Combined	
					*P*	OR (95%CI)	*P*	OR (95%CI)
CPED1, WNT16	rs2707520	Dominant	CC			1.00		1.00
			CA+AA		0.067	0.698 (0.475-1.026)	1.39E-03	0.566 (0.400-0.803)
	rs2707521	Dominant	CC			1.00		1.00
			CT+TT		0.089	0.717 (0.488-1.052)	0.002	0.580 (0.410-0.821)
VPS53	rs61644407	Dominant	AA			1.00		1.00
			AC+CC		0.062	1.435 (0.982-2.096)	1.42E-03	1.750 (1.241-2.468)
WWOX	rs75559202	Dominant	CC			1.00		1.00
			CG+GG		0.079	1.510 (0.953-2.390)	0.004	1.846 (1.223-2.785)
SERPINA4	rs5510	Recessive	CC+CT			1.00		1.00
			TT		0.007	0.289 (0.116-0.717)	0.002	0.277 (0.121-0.635)
KIDINS220	rs1044280	Recessive	CC+CA			1.00		1.00
			AA		0.556	0.720 (0.241-2.153)	0.224	1.722 (0.716-4.140)
AOAH	rs2228410	Overdominant	CC+TT			1.00		1.00
			CT		0.024	0.650 (0.448-0.944)	0.009	0.638 (0.455-0.896)
IQGAP2	rs2455230	Dominant	GG			1.00		1.00
			GC+CC		0.008	0.577 (0.384-0.866)	8.90E-04	0.535 (0.370-0.774)
HCG22	rs2523855	Dominant	GG			1.00		1.00
			GC+CC		0.009	0.606 (0.415-0.884)	0.157	0.781 (0.555-1.100)
ZBTB46	rs2281929	Dominant	TT			1.00		1.00
			TC+CC		7.75E-05	0.466 (0.319-0.680)	1.18E-06	0.423 (0.299-0.599)

SNP, single nucleotide polymorphism; OR, odds ratio; CI, confidence interval.

### rs2281929 TT Carriers and rs2455230 GG Carriers Had Lower Fold Rises of Antibodies

**Figure 3:**
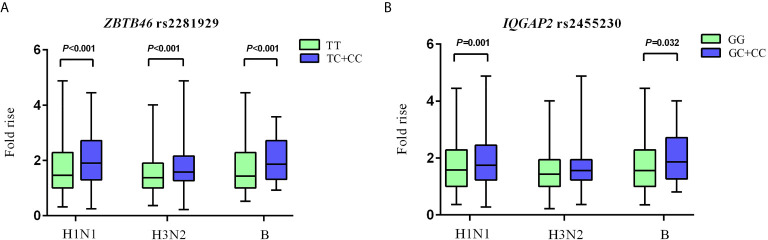
Comparison of antibody fold rises against different vaccine strains. **(A)** The comparison of antibody fold rises between *ZBTB46* rs2281929 TT genotype and TC+CC genotype to H1N1, H3N2 and B. **(B)** The comparison of antibody fold rises between *IQGAP2* rs2455230 GG genotype and GC+CC genotype to H1N1, H3N2 and B.

For *ZBTB46* rs2281929 TT genotype carriers, the antibody fold rises against H1N1, H3N2, and B were all significantly lower than that of subjects with TC + CC genotype (*P* < 0.001). Compared with *IQGAP2* rs2455230 GC + CC carriers, GG carriers had lower antibody fold rises to H1N1 (*P* = 0.001) and B (*P* = 0.032). The GG genotype of rs2455230 tended to be correlated with lower antibody fold rises (*P* = 0.096) against H3N2, but the difference was not statistically significant. (**Figure 3** and **Supplementary Table 8**).

### Verification Study for Nine Previously Reported SNPs

Nine SNPs from published studies were verified in our combined group. All SNPs tested had a call rate ≥95% and conformed to HWE (*P* > 0.001). The allelic and genotypic frequencies of the nine SNPs are listed in **Supplementary Table 9**. **Supplementary Table 10** presents other information of these variants. We observed that the allelic and genotypic distributions of *IL-12B* rs3212227 and *IL-1R1* rs3732131 were significantly different between LR group and responder group (*P* < 0.05). In multivariate logistic regression analysis adjusted for gender and age, compared with the TT genotype of *IL-12B* rs3212227, the TG + GG genotype tended to be correlated with a higher risk of low responsiveness to influenza vaccination (*P* = 0.016, OR = 1.614, 95%CI = 1.093–2.385). The AG + GG genotype of *IL-1R1* rs3732131 tended to be associated with a better immune response to influenza vaccine compared with the AA genotype (*P* = 0.014, OR = 0.644, 95%CI = 0.454–0.913). However, after the Bonferroni correction, no statistical difference was found in any SNP (*P* < 1.32E-03) (**Table 4**).

**Table 4:** Multivariate logistic regression analysis of 9 SNPs from published studies.

Reference	Gene	SNPs	Function	Genetic Model	Genotype	*P*	OR (95%CI)
Cummins NW et al. (16)	HMOX1	rs743811	2.8kb 3’ of HMOX1	Dominant	CC		1.00
					CT+TT	0.505	1.122 (0.800-1.572)
	HMOX2	rs2160567	intronic	Overdominant	TT+CC		1.00
					TC	0.957	1.009 (0.722-1.412)
Poland GA et al. (6)	IL-6	rs1800796	5’-UTR	Dominant	CC		1.00
					CG+GG	0.167	1.268 (0.905-1.776)
	IL-12B	rs3212227	3’-UTR	Dominant	TT		1.00
					TG+GG	0.016	1.614 (1.093-2.385)
	IL-1R1	rs3732131	3’-UTR	Dominant	AA		1.00
					AG+GG	0.014	0.644 (0.454-0.913)
	IL-10RB	rs3171425	3’-UTR	Overdominant	GG+AA		1.00
					GA	0.490	1.127 (0.803-1.581)
	IL-10RA	rs4252249	synonymous	Overdominant	GG+AA		1.00
					GA	0.179	1.449 (0.843-2.489)
	IL-1RN	rs315952	synonymous	Dominant	CC		1.00
					CT+TT	0.112	0.753 (0.531-1.068)
Egli A et al. (8)	IL-28B	rs8099917	7.5kb 5’ of IL28B	Overdominant	TT+GG		1.00
					TG	0.323	1.334 (0.753-2.363)

SNP, single nucleotide polymorphism; OR, odds ratio; CI, confidence interval.

## Discussion

Immune response to vaccine is a complicated process, which requires regulations from a series of molecules. A variant in any of these molecules may alter the level of the antibody response. Identifying these variants will make great contributions to improve the individualized influenza vaccination programs.

In this study, we focused on identifying immune-related genetic factors associated with the responsiveness to influenza vaccination. In the discovery stage, in order to identify the variants that are most likely to influence the antibody response, we selected subjects with extreme phenotypes. In the GWAS, regardless of the type of vaccine strain, only individuals whose immune responses to all vaccine strains reached neither the seroconversion rate nor the seroprotection rate can be included in the case group. Subjects whose HAI titers reached both the seroconversion rate and the seroprotection rate to all vaccine strains can be included in the control group. In the verification stage, in order to obtain a larger sample size and enhance the statistic power, we appropriately broadened the inclusion criteria of subjects and removed the criteria of seroprotection rate. Compared with the seroprotection rate, the seroconversion rate is a better index to reflect the level of immunity because the baseline antibody level before immunization was taken into consideration. Finally, two SNPs including *ZBTB46* rs2281929 and *IQGAP2* rs2455230 were found to be significantly correlated with the serological response to influenza vaccine. According to the MAF and OR of these two significant SNPs, our sample size was sufficient with a statistical power of 0.80 (**Supplementary Table 11**).

ZBTB46 is a zinc finger and BTB domain-containing transcription factor, which is selectively expressed by classical antigen presenting dendritic cells (cDCs) and their committed progenitors (17, 18). The N-terminal BTB domain is responsible for mediating protein–protein interactions, while the C-terminal is for DNA binding. Transcription of target genes can be initiated or inhibited (often inhibited) in collaboration with chromatin remodeling complexes recruited by the BTB domain (19). cDCs are immune accessory cells critical for adaptive responses against pathogens and play an important role in the process of antigen presentation (20). Researchers identified that ZBTB46 may inhibit the activation of cDCs during steady-state conditions and maintain cDCs in this quiescent state (21). When exploring the target genes of ZBTB46, Meredith et al. found that ZBTB46 binds a variety of genes in cDCs, including MHC II antigens, and behaves as a negative regulator of cDC gene expression (22). In contrast to ZBTB46, Creb1 is an activator of MHC and may compete with ZBTB46 for MHC promoter binding (23, 24). In mouse model, Meredith et al. also observed that ZBTB46-deficient cDCs expressed higher Creb1 transcript levels than wild-type controls. Compared with wild-type littermates, cDCs also expressed up to twofold higher MHC II levels in ZBTB46-deficient mice by flow cytometry. Besides, the deletion of this gene may also altered the proportions of CD4^+^ and CD8^+^ cDCs in the spleen (22). Therefore, considering the important regulatory effect of ZBTB46 on cDCs, the relationship between ZBTB46 genetic variants and vaccine immunity deserves further investigation.

*ZBTB46* rs2281929 is a missense and the most significant variant in our validation study. Some studies showed that rs2281929 may be associated with the telomere length (25, 26) and glioblastoma (27), but the correlation is not strong. No previous studies have reported the relationship between rs2281929 and vaccine immunity. Our study showed that compared with the TT genotype, the TC + CC genotype of *ZBTB46* rs2281929 was significantly associated with better responsiveness to influenza vaccination in both qualitative and quantitative analyses. The change in allele T to C results in a change in Threonine to Alanine. Therefore, we hypothesized that the change from Threonine to Alanine in this site may downregulate the activation of ZBTB46, reactivated the expression of cDCs as well as the antigen presentation, and finally induce a better vaccine immune response. Besides, rs2281929 is also a splicing site according to the prediction from SNPinfo (**Supplementary Table 12**). Further study should be performed to analyze the relationship between this variant and the function of the gene.

IQGAP2 (IQ Motif Containing GTPase Activating Protein 2) is a 180 kDa cytoplasmic multidomain scaffolding protein that belongs to the IQGAPs family. This family consists of three highly homologous members, IQGAP1, IQGAP2, and IQGAP3 (28). IQGAP proteins may play a role in regulating various cellular processes, including cytokinesis, cell migration and proliferation, intracellular signaling, vesicle trafficking, as well as cytoskeletal dynamics (29). Currently, many studies have been conducted to evaluate the relationship between IQGAP2 and cancers. And most of them reported that the mRNA expression of IQGAP2 was negatively associated with a variety of cancers, including hepatocellular carcinoma, gastric cancer, bladder cancer, breast cancer, kidney cancer, lung cancer, and so on, indicating the potential of IQGAP2 being a tumor suppressor (30–33). Results of these studies also suggested that the expression of this gene could be a biomarker for the prognosis of cancers. In addition to the role as a tumor suppressor, IQGAP2 is also found to be a novel interferon-alpha (IFN-α) antiviral effector gene and may play a role in regulating interferon stimulated genes (ISG) through the NF-*κ*B pathway unconventionally. IFN is a regulatory of many biological processes, such as antiviral responses and immune responses. ISGs are the primary effectors of the IFN antiviral response. IQGAP2 may physically interact with RelA (a subunit of the NF-*κ*B transcription factor, also known as p65) downstream of the IFN binding site and act cooperatively to mediate the antiviral response of IFN (34). IFITM3 is a kind of ISG. Lei et al. reported that the deletion of IFITM3 attenuated the antibody response to influenza vaccination (35). However, no association has been investigated between IQGAP2 and IFITM3. Ghaleb et al. found that knockout of IQGAP2 gene in mice has a protective effect on dextran sulfate sodium (DSS)-induced colitis in mice. In IQGAP2-deficient mice, the p65 subunit of NF-*κ*B diminished, and the levels of macrophages and neutrophils decreased in comparison to those of wild-type mice (36). Collectively, all these results point to a potential role of IQGAP2 in the immune system. It is worthy to analyze the function of this gene in the antibody response.

As the second significant SNP identified from our study, *IQGAP2* rs2455230 is a missense variant that may result in a change of Leucine to Phenylalanine from the change of G allele to C allele. In addition, the potential of rs2455230 acting as a splicing variant is also predicted from the SNPinfo (**Supplementary Table 12**). Our study observed that rs2455230 GC + CC genotype was associated with a lower risk of low responsiveness to influenza vaccine compared with the GG genotype. Thus, we presumed that the change from Leucine to Phenylalanine in this site may enhance the serological response to vaccine through the mediation of IQGAP2.

According to published studies on the relationship between genetic variants and the immune response to influenza vaccine, nine significant SNPs were validated in our study but no correlation was found (*P* < 1.32E-03). In **Supplementary Table 10**, we summarized the characteristics of three published studies mentioned in our verification analysis. We presumed that the reasons why our study results differ from previously published results are mainly caused by differences in ethnicity and study design. Whether these nine SNPs have any impact on the antibody response to influenza vaccine remains to be confirmed.

The limitations of our study were summarized as follows. Firstly, our GWA stage included only 111 subjects, which is a small sample size and may lead to false-negative results and the inability to identify infrequent variants. Secondly, only gender and age were included as confounding factors in the multivariate analysis. Further study should collect and contain more factors that may influence the immunogenicity of influenza vaccines. Finally, we observed that *ZBTB46* rs2281929 and *IQGAP2* rs2455230 are associated with the antibody response to influenza vaccine. However, the exact role of these two genes and SNPs in vaccine immunity remains unclear and requires further exploration by experimental animal models. To deal with the limitations, a series of processes were performed. In GWAS, we adopted strict inclusion criteria of subjects and a standardized procedure of quality control. In the stage of SNP selection, we carefully screened the variants considering the gene function. Then, a replication study with a larger sample size was performed to validate the results and filter out the false negative variants. We also corrected the *P*-value statistically to control the probability of making mistakes.

To the best of our knowledge, this is the first GWAS conducted to identify genetic variants associated with the immune response to influenza vaccination in the Chinese population. In conclusion, we have identified two novel candidate missense variants, rs2281929 and rs2455230, located in the exon region of ZBTB46 and IQGAP2 respectively. Further studies will be carried out to explore the mechanism of the regulatory function of these two genes in vaccine immunity.

## Data Availability Statement

The raw sequence data reported in this paper have been deposited in the China National GeneBank DataBase (CNGBdb), under accession number CNP0001871 that are publicly accessible at https://db.cngb.org/search/project/CNP0001871/.

## Ethics Statement

The studies involving human participants were reviewed and approved by the Ethics Review Committee of the National Institute for Viral Disease Control and Prevention (NIVDC, assurance number, 200916), and written informed consent was obtained from all volunteers. Written informed consent to participate in this study was provided by the participants’ legal guardian/next of kin.

## Author Contributions

YS and DW designed the study. SW and HW managed the cohort. SW, ML and SZ performed the experiments. YC and WH contributed in collecting the samples and epidemiology data. QL and SW processed the data. SW and YS are responsible for all the analyses and write this paper. All authors contributed to the article and approved the submitted version.

## Funding

This study was supported by the Shenzhen science and technology program (Grant number: kqtd20180411143323605) and The National Natural Science Foundation of China (Grant number: 82041043).

## Conflict of Interest

The authors declare that the research was conducted in the absence of any commercial or financial relationships that could be construed as a potential conflict of interest.
