# Supplementary material for: Identification of Two Novel Candidate Genetic Variants Associated With the Responsiveness to Influenza Vaccination
Source: Front Immunol. 2021 Jul 1;12:664024. doi: 10.3389/fimmu.2021.664024 (PMC8281270; doi:10.3389/fimmu.2021.664024)
Supplement: Supplementary file 1 [file DataSheet_1.docx]

| **Supplementary table 1.** Basic information of whole-genome sequencing. | | |
| --- | --- | --- |
| Criteria | Range | ‾x ± S |
| Clean bases (Gb) | 101.2~158.2 | 121.92±9.27 |
| Mapping rate (%) | 99.42~99.93 | 99.87±0.07 |
| Unique rate (%) | 91.19~95.14 | 93.74±0.63 |
| Duplicate rate (%) | 1.09~4.61 | 2.06±0.55 |
| Mismatch rate (%) | 0.39~0.93 | 0.58±0.10 |
| Average sequencing depth | 32.53~50.39 | 39.09±3.39 |
| Coverage (%) | 95.47~99.9% | 96.49±1.68 |
| Coverage at least 4X (%) | 95.17~98.9% | 96.23±1.69 |
| Coverage at least 10X (%) | 93.80~97.5% | 95.76±1.71 |
| Coverage at least 20X (%) | 86.96~95.2% | 93.26±2.55 |

| **Supplementary table 2.** Quality control of variants. | | | |
| --- | --- | --- | --- |
| Filter | Criteria | SNP Removed | Indel Removed |
| Depth for minor allele | Minor allele average depth<4 | 738,300 | 150,018 |
| Depth for site | Case or control average depth<8 | 697,648 | 112,459 |
| VQSR | Filtered | 1,404,168 | 342,921 |
| 8X depth fraction of site | Case or control 8X rate<0.9 | 1,391,070 | 335,947 |
| Homopolymer | Homopolymer run≥6 | 2,369,950 | 1,741,208 |
| Allelic balance in heterozygotes | Allelic balance < 0.25 or > 0.75 | 1,480,879 | — |
| Mapping quality score | Root mean square <30 | 709,238 | 151,532 |
| Strand bias | Fisher test P-value <10^-5^ and OR>3 | 507,180 | 53,494 |
| Fraction of unbias allelic balance in heterozygotes | The fraction of unbias allelic balance < 0.9 | 2,524,958 | — |
| 4X depth fraction for minor allele in heterozygotes | 4X depth fraction < 0.9 | 2,217,824 | 1,344,752 |
| Hardy-Weinberg disequilibrium | P < 10^-6^ | 21,291 | 1,256 |
| All above filters | Any one | 4,958,019 | 2,136,586 |
| Retained |  | 12,271,151 | 742,844 |

| **Supplementary table 3.** Vaccine strains recommended by WHO in the northern hemisphere. | | | |
| --- | --- | --- | --- |
| Flu season | H1N1 | H3N2 | BV/BY |
| 2009-2010 | A/Brisbane/59/2007 (H1N1) | A/Brisbane/10/2007 (H3N2) | B/Brisbane/60/2008(B/Victoria) |
| 2010-2011 | A/California/7/2009 (H1N1) | A/Perth/16/2009 (H3N2) | B/Brisbane/60/2008(B/Victoria) |
| 2011-2012 | A/California/7/2009 (H1N1) | A/Perth/16/2009 (H3N2) | B/Brisbane/60/2008(B/Victoria) |
| 2012-2013 | A/California/7/2009 (H1N1) | A/Victoria/361/2011 (H3N2) | B/Wisconsin/1/2010(B/Yamagata) |
| 2013-2014 | A/California/7/2009 (H1N1) | A/Texas/50/2012 (H3N2) | B/Massachusetts/2/2012(B/Yamagata) |
| 2014-2015 | A/California/7/2009 (H1N1) | A/Texas/50/2012 (H3N2) | B/Massachusetts/2/2012(B/Yamagata) |
| 2015-2016 | A/California/7/2009 (H1N1) | A/Switzerland/9715293/2013 (H3N2) | B/Phuket/3073/2013(B/Yamagata) |
| 2016-2017 | A/California/7/2009 (H1N1) | A/Hong Kong/4801/2014 (H3N2) | B/Brisbane/60/2008(B/Victoria) |
| 2017-2018 | A/Michigan/45/2015 (H1N1) | A/Hong Kong/4801/2014 (H3N2) | B/Brisbane/60/2008(B/Victoria) |
| 2018-2019 | A/Michigan/45/2015 (H1N1) | A/Singapore/INFIMH-16-0019/2016 (H3N2) | B/Colorado/06/2017 (B/Victoria) |
| 2019-2020 | A/Brisbane/02/2018 (H1N1) | A/Kansas/14/2017 (H3N2) | B/Colorado/06/2017 (B/Victoria) |

| **Supplementary table 4.** Highly significant variants (*P*<1×10^-5^) identified from genome-wide association study. | | | | | | | | | | | | | | |
| --- | --- | --- | --- | --- | --- | --- | --- | --- | --- | --- | --- | --- | --- | --- |
| Number | Chr | SNP | Position | | Nearest Gene | Other nearby gene | | Minor  allele | Major  allele | Function | MAF | | *P* ^a^ | OR (95%CI) |
|  |  |  |  |  |  |  |  |  |  |  | Case | Control |  |  |
| 1 | 18 | rs2847111 | 13501672 | | LDLRAD4 |  | | A | G | intronic | 0.029 | 0.305 | 8.82E-07 | 0.069(0.016-0.294) |
| 2 | 10 | rs3997977 | 77424506 | | KCNMA1 |  | GTTCA | | G | intronic | 0.515 | 0.195 | 2.70E-06 | 4.384(2.357-8.154) |
| 3 | 1 | rs3118200 | 33751928 | | CSMD2 |  | | A | T | intronic | 0.044 | 0.312 | 3.60E-06 | 0.102(0.031-0.341) |
| 4 | 6 | rs112642128 | | 41755261 | PGC | FRS3 | | C | T | intergenic | 0.059 | 0.331 | 3.91E-06 | 0.126(0.044-0.366) |
| 5 | 18 | rs3216103 | 13501275 | | LDLRAD4 |  | | TAG | DEL | intronic | 0.059 | 0.331 | 3.91E-06 | 0.126(0.044-0.366) |
| 6 | 18 | rs2555395 | 13504552 | | LDLRAD4 |  | | C | G | intronic | 0.059 | 0.331 | 3.91E-06 | 0.126(0.044-0.366) |
| 7 | 5 | rs4921534 | 159895216 | | ADRA1B | LINC01847 | | G | A | intergenic | 0.309 | 0.065 | 4.56E-06 | 6.434(2.828-14.64) |
| 8 | 5 | rs2195926 | 159895916 | | ADRA1B | LINC01847 | | A | G | intergenic | 0.309 | 0.065 | 4.56E-06 | 6.434(2.828-14.64) |
| 9 | 10 | rs287186 | 77422240 | | KCNMA1 |  | | G | T | intronic | 0.515 | 0.201 | 5.58E-06 | 4.208(2.269-7.803) |
| 10 | 10 | rs158421 | 77422276 | | KCNMA1 |  | | C | T | intronic | 0.515 | 0.201 | 5.58E-06 | 4.208(2.269-7.803) |
| 11 | 10 | rs287187 | 77423073 | | KCNMA1 |  | | A | T | intronic | 0.515 | 0.201 | 5.58E-06 | 4.208(2.269-7.803) |
| 12 | 10 | rs660465 | 77425353 | | KCNMA1 |  | | C | T | intronic | 0.515 | 0.201 | 5.58E-06 | 4.208(2.269-7.803) |
| 13 | 10 | rs660940 | 77425482 | | KCNMA1 |  | | G | T | intronic | 0.515 | 0.201 | 5.58E-06 | 4.208(2.269-7.803) |
| 14 | 10 | rs674840 | 77426285 | | KCNMA1 |  | | A | T | intronic | 0.515 | 0.201 | 5.58E-06 | 4.208(2.269-7.803) |
| 15 | 10 | rs595386 | 77428960 | | KCNMA1 |  | | G | A | intronic | 0.515 | 0.201 | 5.58E-06 | 4.208(2.269-7.803) |
| 16 | 10 | rs610097 | 77429012 | | KCNMA1 |  | | G | A | intronic | 0.515 | 0.201 | 5.58E-06 | 4.208(2.269-7.803) |
| 17 | 10 | rs676267 | 77429408 | | KCNMA1 |  | | C | T | intronic | 0.515 | 0.201 | 5.58E-06 | 4.208(2.269-7.803) |
| 18 | 10 | rs583728 | 77430512 | | KCNMA1 |  | | C | T | intronic | 0.515 | 0.201 | 5.58E-06 | 4.208(2.269-7.803) |
| 19 | 10 | rs584141 | 77430596 | | KCNMA1 |  | | G | T | intronic | 0.515 | 0.201 | 5.58E-06 | 4.208(2.269-7.803) |
| 20 | 10 | rs585895 | | 77430992 | KCNMA1 |  | | A | T | intronic | 0.515 | 0.201 | 5.58E-06 | 4.208(2.269-7.803) |
| 21 | 6 | rs62396738 | | 41761259 | PGC | FRS3 | | T | C | intergenic | 0.059 | 0.325 | 6.76E-06 | 0.130(0.045-0.377) |
| 22 | 6 | rs12661968 | | 41761668 | PGC | FRS3 | | C | T | intergenic | 0.059 | 0.325 | 6.76E-06 | 0.130(0.045-0.377) |
| 23 | 12 | rs1567584 | | 51270520 | SMAGP | BIN2 | | A | C | upstream | 0.265 | 0.045 | 7.13E-06 | 7.560(2.982-19.160) |
| 24 | 12 | rs1567583 | | 51270557 | SMAGP | BIN2 | | T | G | upstream | 0.265 | 0.045 | 7.13E-06 | 7.560(2.982-19.160) |
| 25 | 12 | rs79104915 | | 51270727 | SMAGP | BIN2 | | G | C | upstream | 0.265 | 0.045 | 7.13E-06 | 7.560(2.982-19.160) |
| 26 | 12 | rs76878808 | | 51271480 | SMAGP | BIN2 | | A | G | intergenic | 0.265 | 0.045 | 7.13E-06 | 7.560(2.982-19.160) |
| 27 | 12 | rs117626990 | | 51271803 | SMAGP | BIN2 | | T | G | intergenic | 0.265 | 0.045 | 7.13E-06 | 7.560(2.982-19.160) |
| 28 | 12 | rs17125583 | | 51272022 | SMAGP | BIN2 | | C | T | intergenic | 0.265 | 0.045 | 7.13E-06 | 7.560(2.982-19.160) |
| 29 | 12 | rs17125588 | | 51272302 | SMAGP | BIN2 | | T | C | intergenic | 0.265 | 0.045 | 7.13E-06 | 7.560(2.982-19.160) |
| 30 | 12 | rs11832769 | | 51272769 | SMAGP | BIN2 | | T | C | intergenic | 0.265 | 0.045 | 7.13E-06 | 7.560(2.982-19.160) |
| 31 | 12 | rs199570770 | | 51272918 | SMAGP | BIN2 | | C | CT | intergenic | 0.265 | 0.045 | 7.13E-06 | 7.560(2.982-19.160) |
| 32 | 12 | rs140274828 | | 51274319 | SMAGP | BIN2 | | T | C | intergenic | 0.265 | 0.045 | 7.13E-06 | 7.560(2.982-19.160) |
| 33 | 12 | rs79074013 | | 51277504 | SMAGP | BIN2 | | C | T | intergenic | 0.265 | 0.045 | 7.13E-06 | 7.560(2.982-19.160) |
| 34 | 12 | rs117440918 | | 51278765 | SMAGP | BIN2 | | T | C | intergenic | 0.265 | 0.045 | 7.13E-06 | 7.560(2.982-19.160) |
| 35 | 12 | rs140744411 | | 51279868 | SMAGP | BIN2 | | T | C | intergenic | 0.265 | 0.045 | 7.13E-06 | 7.560(2.982-19.160) |
| 36 | 12 | rs139717220 | | 51288941 | BIN2 |  | | A | G | intronic | 0.265 | 0.045 | 7.13E-06 | 7.560(2.982-19.160) |
| 37 | 12 | rs117335549 | | 51290202 | BIN2 |  | | A | G | intronic | 0.265 | 0.045 | 7.13E-06 | 7.560(2.982-19.160) |
| 38 | 12 | rs79771928 | | 51293739 | BIN2 |  | | A | G | intronic | 0.265 | 0.045 | 7.13E-06 | 7.560(2.982-19.160) |
| 39 | 12 | rs78498474 | | 51294207 | BIN2 |  | | G | A | intronic | 0.265 | 0.045 | 7.13E-06 | 7.560(2.982-19.160) |
| 40 | 12 | rs141943976 | | 51294974 | BIN2 |  | | T | C | intronic | 0.265 | 0.045 | 7.13E-06 | 7.560(2.982-19.160) |
| 41 | 13 | rs59711400 | | 23487181 | LINC00327 |  | | A | G | ncRNA | 0.221 | 0.026 | 7.71E-06 | 10.610(3.372-33.400) |
| 42 | 7 | rs2707521 | 121300382 | | CPED1 | WNT16 | | T | C | intergenic | 0.206 | 0.526 | 8.09E-06 | 0.234(0.120-0.456) |
| 43 | 7 | rs6980043 | 121306700 | | CPED1 | WNT16 | | T | C | intergenic | 0.206 | 0.526 | 8.09E-06 | 0.234(0.120-0.456) |
| 44 | 7 | rs2707520 | 121311090 | | CPED1 | WNT16 | | A | C | intergenic | 0.206 | 0.526 | 8.09E-06 | 0.234(0.120-0.456) |
| 45 | 7 | rs10231005 | 121312528 | | CPED1 | WNT16 | | A | C | intergenic | 0.206 | 0.526 | 8.09E-06 | 0.234(0.120-0.456) |
| Chr, chromosome; SNP, single nucleotide polymorphism; MAF, minor allele frequency; OR, odds ratio; CI, confidence interval. | | | | | | | | | | | | | | |

| **Supplementary table 5.** Gene-based association analysis in group 1. | | | | | | | | |
| --- | --- | --- | --- | --- | --- | --- | --- | --- |
| Mutation set | Chromosome | Gene | Number of carriers | | Number of non-carriers | | *P*(Fisher) | OR(95%CI) |
|  |  |  | Low responders | Responders | Low responders | Responders |  |  |
| Deleterious (Broad) | chr16 | NOB1 | 5 | 0 | 29 | 77 | 2.17E-03 | inf(2.24-inf) |
|  | chr19 | OR7D2 | 5 | 0 | 29 | 77 | 2.17E-03 | inf(2.24-inf) |
|  | chr16 | RPL3L | 9 | 4 | 25 | 73 | 2.71E-03 | 6.44(1.62-31.19) |
|  | chr10 | CC2D2B | 8 | 3 | 26 | 74 | 3.15E-03 | 7.43(1.63-46.69) |
|  | chr11 | ALKBH8 | 6 | 1 | 28 | 76 | 3.21E-03 | 15.84(1.80-755.39) |
|  | chr1 | CDC42BPA | 6 | 1 | 28 | 76 | 3.21E-03 | 15.84(1.80-755.39) |
|  | chr10 | FRMPD2 | 7 | 2 | 27 | 75 | 3.40E-03 | 9.49(1.67-99.16) |
|  | chr3 | SLC25A26 | 7 | 2 | 27 | 75 | 3.40E-03 | 9.49(1.67-99.16) |
|  | chr10 | ITIH5 | 10 | 6 | 24 | 71 | 6.28E-03 | 4.85(1.42-18.09) |
|  | chr7 | DNAH11 | 23 | 30 | 11 | 47 | 7.20E-03 | 3.24(1.30-8.51) |
|  | chr1 | DISP3 | 8 | 4 | 26 | 73 | 7.31E-03 | 5.51(1.34-27.18) |
|  | chr5 | ADRA1B | 4 | 0 | 30 | 77 | 7.74E-03 | inf(1.58-inf) |
|  | chr15 | MFAP1 | 4 | 0 | 30 | 77 | 7.74E-03 | inf(1.58-inf) |
|  | chr1 | MKNK1 | 4 | 0 | 30 | 77 | 7.74E-03 | inf(1.58-inf) |
|  | chr6 | PHF1 | 4 | 0 | 30 | 77 | 7.74E-03 | inf(1.58-inf) |
|  | chr1 | RERE | 4 | 0 | 30 | 77 | 7.74E-03 | inf(1.58-inf) |
|  | chr16 | RPGRIP1L | 4 | 0 | 30 | 77 | 7.74E-03 | inf(1.58-inf) |
|  | chr11 | SLC22A9 | 4 | 0 | 30 | 77 | 7.74E-03 | inf(1.58-inf) |
|  | chr17 | TNK1 | 4 | 0 | 30 | 77 | 7.74E-03 | inf(1.58-inf) |
|  | chr8 | PKHD1L1 | 14 | 13 | 20 | 64 | 8.50E-03 | 3.4(1.26-9.38) |
|  | chr2 | PXDN | 7 | 3 | 27 | 74 | 8.75E-03 | 6.27(1.32-40.27) |
|  | chr3 | SLC25A26 | 7 | 3 | 27 | 74 | 8.75E-03 | 6.27(1.32-40.27) |
|  | chr2 | PLB1 | 0 | 13 | 34 | 64 | 8.83E-03 | 0(0.00-0.67) |
|  | chr9 | DOCK8 | 9 | 5 | 25 | 72 | 9.88E-03 | 5.09(1.38-21.29) |
|  | chr10 | ITIH5 | 9 | 5 | 25 | 72 | 9.88E-03 | 5.09(1.38-21.29) |
|  | chr19 | ANKRD27 | 6 | 2 | 28 | 75 | 9.89E-03 | 7.86(1.31-84.29) |
|  | chr4 | FAM47E | 6 | 2 | 28 | 75 | 9.89E-03 | 7.86(1.31-84.29) |
| Deleterious (Strict) | chr15 | AGBL1 | 8 | 4 | 26 | 73 | 7.31E-03 | 5.51 (1.34-27.18) |
|  | chr4 | FAM47E | 6 | 2 | 28 | 75 | 9.89E-03 | 7.86 (1.31-84.29) |
| “Deleterious (Broad)” as defined by nonsense, splice-site, indel frameshift, and missense annotated as deleterious by at least one of the five protein prediction algorithms of LRT score, Mutation Taster, PolyPhen-2 HumDiv, PolyPhen-2 HumVar and SIFT; “Deleterious (Strict)” as defined by nonsense, splice-site, indel frameshift, and missense annotated as deleterious by all five protein prediction algorithms. | | | | | | | | |

| **Supplementary table 6.** Univariant analysis of 29 candidate variants in group 2. | | | | | | | | | | | | | |
| --- | --- | --- | --- | --- | --- | --- | --- | --- | --- | --- | --- | --- | --- |
| Gene | Variants | Genotype/  Allele |  | Low-responders (%) | | Responders (%) | | *P* | HWE- *P* | | MAF | MAF(CHB) | Call rate (%) |
| LDLRAD4 | rs2847111 | GG |  | 111(57.8) | 151(53.0) | | | 0.118 | 0.148 | | 0.251 | 0.204 | 99.17 |
|  |  | GA |  | 76(39.6) | 115(40.3) | | |  |  | |  |  |  |
|  |  | AA |  | 5(2.6) | 19(6.7) | | |  |  | |  |  |  |
|  |  | G |  | 298(77.6) | 417(73.2) | | | 0.120 |  | |  |  |  |
|  |  | A |  | 86(22.4) | 153(26.8) | | |  |  | |  |  |  |
|  | rs3216103 | DEL.DEL |  | 96(50.0) | 134(47.0) | | | 0.682 | 0.142 | | 0.296 | 0.277 | 99.17 |
|  |  | DEL.TAG |  | 84(43.7) | 128(44.9) | | |  |  | |  |  |  |
|  |  | TAG.TAG |  | 12(6.3) | 23(8.1) | | |  |  | |  |  |  |
|  |  | DEL |  | 276(71.9) | 396(69.5) | | | 0.425 |  | |  |  |  |
|  |  | TAG |  | 108(28.1) | 174(30.5) | | |  |  | |  |  |  |
| KCNMA1 | rs287187 | TT |  | 87(45.6) | 141(49.5) | | | 0.665 | 0.474 | | 0.313 | 0.325 | 98.96 |
|  |  | TA |  | 82(42.9) | 116(40.7) | | |  |  | |  |  |  |
|  |  | AA |  | 22(11.5) | 28(9.8) | | |  |  | |  |  |  |
|  |  | T |  | 256(67.0) | 398(69.8) | | | 0.360 |  | |  |  |  |
|  |  | A |  | 126(33.0) | 172(30.2) | | |  |  | |  |  |  |
| BIN2, SMAGP | rs1567584 | CC |  | 153(80.5) | 230(81.0) | | | 0.975^a^ | 0.026 | | 0.107 | 0.073 | 98.54 |
|  |  | CA |  | 33(17.4) | 48(16.9) | | |  |  | |  |  |  |
|  |  | AA |  | 4(2.1) | 6(2.1) | | |  |  | |  |  |  |
|  |  | C |  | 339(89.2) | 508(89.4) | | | 0.912 |  | |  |  |  |
|  |  | A |  | 41(10.8) | 60(10.6) | | |  |  | |  |  |  |
| CPED1, WNT16 | rs2707520 | CC |  | 79(41.5) | 92(32.6) | | | 0.132 | 0.411 | | 0.406 | 0.442 | 98.13 |
|  |  | CA |  | 82(43.2) | 137(48.6) | | |  |  | |  |  |  |
|  |  | AA |  | 29(15.3) | 53(18.8) | | |  |  | |  |  |  |
|  |  | C |  | 240(63.2) | 321(56.9) | | | 0.055 |  | |  |  |  |
|  |  | A |  | 140(36.8) | 243(43.1) | | |  |  | |  |  |  |
|  | rs2707521 | CC |  | 79(41.1) | 93(32.6) | | | 0.150 | 0.660 | | 0.404 | 0.442 | 99.17 |
|  |  | CT |  | 85(44.3) | 140(49.1) | | |  |  | |  |  |  |
|  |  | TT |  | 28(14.6) | 52(18.3) | | |  |  | |  |  |  |
|  |  | C |  | 243(63.3) | 326(57.2) | | | 0.060 |  | |  |  |  |
|  |  | T |  | 141(36.7) | 244(42.8) | | |  |  | |  |  |  |
| GPATCH4 | rs11264505 | AA |  | 179(93.7) | 257(90.5) | | | 0.118^a^ | 0.857 | | 0.042 | 0.092 | 98.75 |
|  |  | AG |  | 11(5.8) | 27(9.5) | | |  |  | |  |  |  |
|  |  | GG |  | 1(0.5) | 0(0.0) | | |  |  | |  |  |  |
|  |  | A |  | 369(96.6) | 541(95.2) | | | 0.310 |  | |  |  |  |
|  |  | G |  | 13(3.4) | 27(4.8) | | |  |  | |  |  |  |
| KDELC1 | rs1047740 | CC |  | 52(27.2) | 82(29.0) | | | 0.914 | 0.493 | | 0.476 | 0.481 | 98.54 |
|  |  | CT |  | 94(49.2) | 135(47.7) | | |  |  | |  |  |  |
|  |  | TT |  | 45(23.6) | 66(23.3) | | |  |  | |  |  |  |
|  |  | C |  | 198(51.8) | 299(52.8) | | | 0.764 |  | |  |  |  |
|  |  | T |  | 184(48.2) | 267(47.2) | | |  |  | |  |  |  |
| BCL2L14 | rs879732 | CC |  | 47(24.5) | 83(29.3) | | | 0.485 | 0.628 | | 0.472 | 0.495 | 98.75 |
|  |  | CT |  | 103(53.6) | 139(49.1) | | |  |  | |  |  |  |
|  |  | TT |  | 42(21.9) | 61(21.6) | | |  |  | |  |  |  |
|  |  | C |  | 197(51.3) | 305(53.9) | | | 0.434 |  | |  |  |  |
|  |  | T |  | 187(48.7) | 261(46.1) | | |  |  | |  |  |  |
| VPS53 | rs61644407 | AA |  | 93(49.7) | 162(58.9) | | | 0.073 | 0.405 | | 0.252 | 0.257 | 96.05 |
|  |  | AC |  | 85(45.5) | 96(34.9) | | |  |  | |  |  |  |
|  |  | CC |  | 9(4.8) | 17(6.2) | | |  |  | |  |  |  |
|  |  | A |  | 271(72.5) | 420(76.4) | | | 0.180 |  | |  |  |  |
|  |  | C |  | 103(27.5) | 130(23.6) | | |  |  | |  |  |  |
| WWOX | rs75559202 | CC |  | 147(76.2) | 239(83.6) | | | 0.094^a^ | 0.663 | | 0.103 | 0.107 | 99.58 |
|  |  | CG |  | 44(22.8) | 43(15.0) | | |  |  | |  |  |  |
|  |  | GG |  | 2(1.0) | 4(1.4) | | |  |  | |  |  |  |
|  |  | C |  | 338(87.6) | 521(91.1) | | | 0.079 |  | |  |  |  |
|  |  | G |  | 48(12.4) | 51(8.9) | | |  |  | |  |  |  |
| HDAC9 | rs73683127 | GG |  | 123(65.1) | 191(67.7) | | | 0.696^a^ | 0.323 | | 0.179 | 0.141 | 97.92 |
|  |  | GT |  | 62(32.8) | 83(29.5) | | |  |  | |  |  |  |
|  |  | TT |  | 4(2.1) | 8(2.8) | | |  |  | |  |  |  |
|  |  | G |  | 308(81.5) | 465(82.4) | | | 0.705 |  | |  |  |  |
|  |  | T |  | 70(18.5) | 99(17.6) | | |  |  | |  |  |  |
| SERPINA4 | rs5510 | CC |  | 103(53.7) | 142(50.3) | | | 0.025 | 0.462 | | 0.276 | 0.325 | 98.54 |
|  |  | CT |  | 83(43.2) | 113(40.1) | | |  |  | |  |  |  |
|  |  | TT |  | 6(3.1) | 27(9.6) | | |  |  | |  |  |  |
|  |  | C |  | 289(75.3) | 397(70.4) | | | 0.100 |  | |  |  |  |
|  |  | T |  | 95(24.7) | 167(29.6) | | |  |  | |  |  |  |
| BIN2 | rs3210837 | CC |  | 137(71.3) | 205(71.9) | | | 0.955 | 0.006 | | 0.164 | 0.146 | 99.17 |
|  |  | CT |  | 47(24.5) | 67(23.5) | | |  |  | |  |  |  |
|  |  | TT |  | 8(4.2) | 13(4.6) | | |  |  | |  |  |  |
|  |  | C |  | 321(83.6) | 477(83.7) | | | 0.970 |  | |  |  |  |
|  |  | T |  | 63(16.4) | 93(16.3) | | |  |  | |  |  |  |
| NLRP8 | rs306507 | TT |  | 119(62.0) | 188(66.4) | | | 0.434 | 0.126 | | 0.203 | 0.170 | 98.75 |
|  |  | TC |  | 64(33.3) | 79(27.9) | | |  |  | |  |  |  |
|  |  | CC |  | 9(4.7) | 16(5.7) | | |  |  | |  |  |  |
|  |  | T |  | 302(78.6) | 455(80.4) | | | 0.512 |  | |  |  |  |
|  |  | C |  | 82(21.4) | 111(19.6) | | |  |  | |  |  |  |
| KIDINS220 | rs1044280 | CC |  | 128(67.0) | 166(58.7) | | | 0.183 | 0.155 | | 0.206 | 0.160 | 98.54 |
|  |  | CA |  | 58(30.4) | 107(37.8) | | |  |  | |  |  |  |
|  |  | AA |  | 5(2.6) | 10(3.5) | | |  |  | |  |  |  |
|  |  | C |  | 314(82.2) | 439(77.6) | | | 0.083 |  | |  |  |  |
|  |  | A |  | 68(17.8) | 127(22.4) | | |  |  | |  |  |  |
| LOX | rs1800449 | CC |  | 126(65.6) | 175(62.1) | | | 0.614 | 0.024 | | 0.214 | 0.194 | 98.54 |
|  |  | CT |  | 56(29.2) | 87(30.8) | | |  |  | |  |  |  |
|  |  | TT |  | 10(5.2) | 20(7.1) | | |  |  | |  |  |  |
|  |  | C |  | 308(80.2) | 437(77.5) | | | 0.315 |  | |  |  |  |
|  |  | T |  | 76(19.8) | 127(22.5) | | |  |  | |  |  |  |
| RNF31 | rs2277484 | GG |  | 127(66.8) | 199(70.1) | | | 0.163^a^ | 0.295 | | 0.167 | 0.112 | 98.54 |
|  |  | GA |  | 56(29.5) | 82(28.9) | | |  |  | |  |  |  |
|  |  | AA |  | 7(3.7) | 3(1.0) | | |  |  | |  |  |  |
|  |  | G |  | 310(81.6) | 480(84.5) | | | 0.236 |  | |  |  |  |
|  |  | A |  | 70(18.4) | 88(15.5) | | |  |  | |  |  |  |
| MAP3K19 | rs16831235 | GG |  | 144(75.0) | 224(78.6) | | | 0.203^a^ | 0.982 | | 0.122 | 0.117 | 99.17 |
|  |  | GA |  | 43(22.4) | 59(20.7) | | |  |  | |  |  |  |
|  |  | AA |  | 5(2.6) | 2(0.7) | | |  |  | |  |  |  |
|  |  | G |  | 331(86.2) | 507(88.9) | | | 0.203 |  | |  |  |  |
|  |  | A |  | 53(13.8) | 63(11.1) | | |  |  | |  |  |  |
| AOAH | rs2228410 | CC |  | 57(30.0) | 71(25.0) | | | 0.078 | 0.972 | | 0.480 | 0.500 | 98.54 |
|  |  | CT |  | 83(43.7) | 154(54.2) | | |  |  | |  |  |  |
|  |  | TT |  | 50(26.3) | 59(20.8) | | |  |  | |  |  |  |
|  |  | C |  | 197(51.8) | 296(52.1) | | | 0.935 |  | |  |  |  |
|  |  | T |  | 183(48.2) | 272(47.9) | | |  |  | |  |  |  |
| IQGAP2 | rs2455230 | GG |  | 68(35.4) | 70(24.8) | | | 0.042 | 0.155 | | 0.476 | 0.471 | 98.54 |
|  |  | GC |  | 80(41.7) | 141(50.0) | | |  |  | |  |  |  |
|  |  | CC |  | 44(22.9) | 71(25.2) | | |  |  | |  |  |  |
|  |  | G |  | 216(56.2) | 281(49.8) | | | 0.052 |  | |  |  |  |
|  |  | C |  | 168(43.8) | 283(50.2) | | |  |  | |  |  |  |
| SIGLEC15 | rs2919643 | TT |  | 87(45.8) | 127(45.0) | | | 0.945 | 0.518 | | 0.332 | 0.379 | 98.13 |
|  |  | TC |  | 82(43.2) | 121(42.9) | | |  |  | |  |  |  |
|  |  | CC |  | 21(11.0) | 34(12.1) | | |  |  | |  |  |  |
|  |  | T |  | 256(67.4) | 375(66.5) | | | 0.778 | 0.471 | |  |  |  |
|  |  | C |  | 124(32.6) | 189(33.5) | | |  |  | |  |  |  |
| PLD2 | rs17854914 | AA |  | 163(84.9) | 248(87.3) | | | 0.387^a^ | 0.802 | | 0.070 | 0.049 | 98.96 |
|  |  | AG |  | 29(15.1) | 34(12.0) | | |  |  | |  |  |  |
|  |  | GG |  | 0(0.0) | 2(0.7) | | |  |  | |  |  |  |
|  |  | A |  | 355(92.4) | 530(93.3) | | | 0.610 |  | |  |  |  |
|  |  | G |  | 29(7.6) | 38(6.7) | | |  |  | |  |  |  |
| MICAL2 | rs3816921 | CC |  | 50(25.9) | 82(28.8) | | | 0.155 | 0.279 | | 0.486 | 0.456 | 99.38 |
|  |  | CG |  | 86(44.6) | 141(49.5) | | |  |  | |  |  |  |
|  |  | GG |  | 57(29.5) | 62(21.7) | | |  |  | |  |  |  |
|  |  | C |  | 186(48.2) | 305(53.5) | | | 0.106 |  | |  |  |  |
|  |  | G |  | 200(51.8) | 265(46.5) | | |  |  | |  |  |  |
| HCG22 | rs2523855 | GG |  | 92(48.2) | 102(35.9) | | | 0.014 | 0.887 | | 0.362 | 0.442 | 98.75 |
|  |  | GC |  | 81(42.4) | 137(48.2) | | |  |  | |  |  |  |
|  |  | CC |  | 18(9.4) | 45(15.9) | | |  |  | |  |  |  |
|  |  | G |  | 265(69.4) | 341(60.0) | | | 0.003 |  | |  |  |  |
|  |  | C |  | 117(30.6) | 227(40.0) | | |  |  | |  |  |  |
| GSTA2 | rs2180314 | GG |  | 89(46.6) | 144(50.7) | | | 0.672 | 0.836 | | 0.301 | 0.291 | 98.75 |
|  |  | GC |  | 83(43.5) | 115(40.5) | | |  |  | |  |  |  |
|  |  | CC |  | 19(9.9) | 25(8.8) | | |  |  | |  |  |  |
|  |  | G |  | 261(68.3) | 403(71.0) | | | 0.387 |  | |  |  |  |
|  |  | C |  | 121(31.7) | 165(29.0) | | |  |  | |  |  |  |
| ZBTB46 | rs2281929 | TT |  | 98(51.1) | 92(32.4) | | 2.47E-04 | | | 0.062 | 0.384 | 0.447 | 98.96 |
|  |  | TC |  | 68(35.4) | 138(48.6) | | |  |  | |  |  |  |
|  |  | CC |  | 26(13.5) | 54(19.0) | | |  |  | |  |  |  |
|  |  | T |  | 264(68.8) | 322(56.7) | | 1.75E-04 | | |  |  |  |  |
|  |  | C |  | 120(31.2) | 246(43.3) | | |  |  | |  |  |  |
| CIITA | rs78108426 | CC |  | 132(68.8) | 194(68.3) | | | 0.856^a^ | 0.023 | | 0.164 | 0.121 | 98.96 |
|  |  | CA |  | 57(29.7) | 87(30.6) | | |  |  | |  |  |  |
|  |  | AA |  | 3(1.5) | 3(1.1) | | |  |  | |  |  |  |
|  |  | C |  | 321(83.6) | 475(83.6) | | | 0.989 |  | |  |  |  |
|  |  | A |  | 63(16.4) | 93(16.4) | | |  |  | |  |  |  |
| SKIL | rs3772173 | TT |  | 119(62.6) | 186(66.0) | | | 0.510 | 0.076 | | 0.204 | 0.194 | 98.13 |
|  |  | TC |  | 62(32.6) | 79(28.0) | | |  |  | |  |  |  |
|  |  | CC |  | 9(4.7) | 17(6.0) | | |  |  | |  |  |  |
|  |  | T |  | 300(78.9) | 451(80.0) | | | 0.704 |  | |  |  |  |
|  |  | C |  | 80(21.1) | 113(20.0) | | |  |  | |  |  |  |
| ^a^ Fisher exact test. SNP, single nucleotide polymorphism; HWE, Hardy-Weinberg equilibrium; MAF, minor allele frequency; OR, odds ratio; CI, confidence interval. | | | | | | | | | | | | | |

| **Supplementary table 7.** Multivariate logistic regression analysis adjusted for age and gender. | | | | | | | | | | | | |
| --- | --- | --- | --- | --- | --- | --- | --- | --- | --- | --- | --- | --- |
| Gene | Variants | | Genetic Model | | Genotype | | Group 2 | | |  | Combined | |
|  |  |  |  |  |  |  | *P* | | OR (95%CI) |  | *P* | OR (95%CI) |
| LDLRAD4 | rs2847111 | | Dominant | | GG | |  | | 1.00 |  |  | 1.00 |
|  |  | |  | | GA+AA | | 0.338 | | 0.833(0.574-1.210) |  | 0.007 | 0.624(0.442-0.879) |
|  | rs3216103 | | Dominant | | DEL.DEL | |  | | 1.00 |  |  |  |
|  |  | |  | DEL.TAG+TAG.TAG | | | | 0.613 | 0.908(0.626-1.317) |  | 0.042 | 0.704(0.502-0.987) |
| KCNMA1 | rs287187 | | Dominant | | TT | |  | | 1.00 |  |  | 1.00 |
|  |  | |  | | TA+AA | | 0.431 | | 1.161(0.801-1.681) |  | 0.006 | 1.609(1.147-2.257) |
| BIN2, SMAGP | rs1567584 | | Dominant | | CC | |  | | 1.00 |  |  | 1.00 |
|  |  | |  | | CA+AA | | 0.912 | | 1.027(0.642-1.642) |  | 0.033 | 1.576(1.038-2.392) |
| CPED1, WNT16 | rs2707520 | | Dominant | | CC | |  | | 1.00 |  |  | 1.00 |
|  |  | |  | | CA+AA | | 0.067 | | 0.698(0.475-1.026) |  | 1.39E-03 | 0.566(0.400-0.803) |
|  | rs2707521 | | Dominant | | CC | |  | | 1.00 |  |  | 1.00 |
|  |  | |  | | CT+TT | | 0.089 | | 0.717(0.488-1.052) |  | 0.002 | 0.580(0.410-0.821) |
| GPATCH4 | rs11264505 | | Dominant | | AA | |  | | 1.00 |  |  | 1.00 |
|  |  | |  | | AG+GG | | 0.219 | | 0.640(0.314-1.304) |  | 0.375 | 1.299(0.729-2.317) |
| KDELC1 | rs1047740 | | Dominant | | CC | |  | | 1.00 |  |  | 1.00 |
|  |  | |  | | CT+TT | | 0.757 | | 1.067(0.706-1.613) |  | 0.273 | 0.813(0.562-1.177) |
| BCL2L14 | rs879732 | | Dominant | | CC | |  | | 1.00 |  |  | 1.00 |
|  |  | |  | | CT+TT | | 0.204 | | 1.314(0.863-2.000) |  | 0.049 | 1.481(1.002-2.189) |
| VPS53 | rs61644407 | | Dominant | | AA | |  | | 1.00 |  |  | 1.00 |
|  |  | |  | | AC+CC | | 0.062 | | 1.435(0.982-2.096) |  | 1.42E-03 | 1.750(1.241-2.468) |
| WWOX | rs75559202 | | Dominant | | CC | |  | | 1.00 |  |  | 1.00 |
|  |  | |  | | CG+GG | | 0.079 | | 1.510(0.953-2.390) |  | 0.004 | 1.846(1.223-2.785) |
| HDAC9 | rs73683127 | | Overdominant | | GG+TT | |  | | 1.00 |  |  | 1.00 |
|  |  | |  | | GT | | 0.331 | | 1.222(0.816-1.831) |  | 0.020 | 1.544(1.071-2.228) |
| SERPINA4 | rs5510 | | Recessive | | CC+CT | |  | | 1.00 |  |  | 1.00 |
|  |  | |  | | TT | | 0.007 | | 0.289(0.116-0.717) |  | 0.002 | 0.277(0.121-0.635) |
| BIN2 | rs3210837 | | Dominant | | CC | |  | | 1.00 |  |  | 1.00 |
|  |  | |  | | CT+TT | | 0.877 | | 1.033(0.685-1.558) |  | 0.107 | 1.353(0.937-1.953) |
| NLRP8 | rs306507 | | Recessive | | TT+TC | |  | | 1.00 |  |  | 1.00 |
|  |  | |  | | CC | | 0.575 | | 0.785(0.337-1.829) |  | 0.445 | 0.727(0.321-1.648) |
| KIDINS220 | rs1044280 | | Recessive | | CC+CA | |  | | 1.00 |  |  | 1.00 |
|  |  | |  | | AA | | 0.556 | | 0.720(0.241-2.153) |  | 0.224 | 1.722(0.716-4.140) |
| LOX | rs1800449 | | Additive | | CC | |  | | 1.00 |  |  | 1.00 |
|  |  | |  | | CT | | 0.636 | | 0.906(0.601-1.365) |  | 0.484 | 1.141(0.789-1.650) |
|  |  | |  | | TT | | 0.297 | | 0.654(0.294-1.454) |  | 0.846 | 0.931(0.452-1.916) |
| RNF31 | rs2277484 | | Recessive | | GG+GA | |  | | 1.00 |  |  | 1.00 |
|  |  | |  | | AA | | 0.074 | | 3.512(0.885-13.928) |  | 0.013 | 4.392(1.373-14.046) |
| MAP3K19 | rs16831235 | | Additive | | GG | |  | | 1.00 |  |  | 1.00 |
|  |  | |  | | GA | | 0.650 | | 1.110(0.708-1.739) |  | 0.164 | 1.332(0.890-1.994) |
|  |  | |  | | AA | | 0.102 | | 4.050(0.758-21.635) |  | 0.018 | 6.904(1.387-34.366) |
| AOAH | rs2228410 | Overdominant | | | CC+TT | |  | | 1.00 |  |  | 1.00 |
|  |  | |  | | CT | | 0.024 | | 0.650(0.448-0.944) |  | 0.009 | 0.638(0.455-0.896) |
| IQGAP2 | rs2455230 | | Dominant | | GG | |  | | 1.00 |  |  | 1.00 |
|  |  | |  | | GC+CC | | 0.008 | | 0.577(0.384-0.866) |  | 8.90E-04 | 0.535(0.370-0.774) |
| SIGLEC15 | rs2919643 | | Recessive | | TT+TC | |  | | 1.00 |  |  | 1.00 |
|  |  | |  | | CC | | 0.645 | | 0.871(0.485-1.565) |  | 0.277 | 0.744(0.437-1.268) |
| PLD2 | rs17854914 | | Dominant | | AA | |  | | 1.00 |  |  | 1.00 |
|  |  | |  | | AG+GG | | 0.436 | | 1.236(0.725-2.109) |  | 0.782 | 0.932(0.568-1.531) |
| MICAL2 | rs3816921 | | Recessive | | CC+CG | |  | | 1.00 |  |  | 1.00 |
|  |  | |  | | GG | | 0.049 | | 1.530(1.001-2.339) |  | 0.165 | 1.321(0.892-1.955) |
| HCG22 | rs2523855 | | Dominant | | GG | |  | | 1.00 |  |  | 1.00 |
|  |  | |  | | GC+CC | | 0.009 | | 0.606(0.415-0.884) |  | 0.157 | 0.781(0.555-1.100) |
| GSTA2 | rs2180314 | | Dominant | | GG | |  | | 1.00 |  |  | 1.00 |
|  |  | |  | | GC+CC | | 0.388 | | 1.177(0.812-1.706) |  | 0.073 | 1.360(0.972-1.904) |
| ZBTB46 | rs2281929 | | Dominant | | TT | |  | | 1.00 |  |  | 1.00 |
|  |  | |  | | TC+CC | 7.75E-05 | | | 0.466(0.319-0.680) |  | 1.18E-06 | 0.423(0.299-0.599) |
| CIITA | rs78108426 | | Overdominant | | CC+AA | |  | | 1.00 |  |  | 1.00 |
|  |  | |  | | CA | | 0.815 | | 0.953(0.636-1.428) |  | 0.198 | 0.781(0.536-1.138) |
| SKIL | rs3772173 | | Overdominant | | TT+CC | |  | | 1.00 |  |  | 1.00 |
|  |  | |  | | TC | | 0.229 | | 1.281(0.856-1.916) |  | 0.054 | 1.437(0.994-2.076) |
| OR, odds ratio; CI, confidence interval. | | | | | | | | | | | | |

| **Supplementary table 8.** Comparison of antibody fold rises against different vaccine strains. | | | | | | | |
| --- | --- | --- | --- | --- | --- | --- | --- |
| Vaccine strain | rs2281929 | | |  | rs2455230 | | |
|  | TT | TC+CC | *P* |  | GG | GC+CC | *P* |
| H1N1 | 1.46(1.00-2.29) | 1.90(1.30-2.72) | <0.001 |  | 1.56(1.00-2.29) | 1.86(1.27-2.72) | 0.001 |
| H3N2 | 1.38(1.00-1.90) | 1.58(1.27-2.16) | <0.001 |  | 1.43(1.00-1.94) | 1.56(1.23-1.94) | 0.096 |
| B | 1.43(1.00-2.29) | 1.86(1.32-2.72) | <0.001 |  | 1.58(1.00-2.29) | 1.75(1.23-2.45) | 0.032 |
| The HAI titers before and after vaccination were transformed to their logarithms. The fold rises with unnormal distribution through Kolmogorov-Smirnov Test were described as the median (IQR) and compared by Mann-Whitney U Test. | | | | | | | |
|  | | | | | | | |

| **Supplementary table 9.** Univariate analysis of 9 SNPs from published reports in the combined group. | | | | | | | | |  |  |
| --- | --- | --- | --- | --- | --- | --- | --- | --- | --- | --- |
| Gene | SNPs | Genotype/  Allele | Low-responders (%) | Responders (%) | *P* | HWE- *P* | MAF | MAF(CHB) | Call rate  (%) |  |
| HMOX1 | rs743811 | CC | 96(42.5) | 164(45.3) | 0.750 | 0.902 | 0.334 | 0.320 | 99.32 |  |
|  |  | CT | 103(45.6) | 160(44.2) |  |  |  |  |  |  |
|  |  | TT | 27(11.9) | 38(10.5) |  |  |  |  |  |  |
|  |  | C | 295(65.3) | 488(67.4) | 0.450 |  |  |  |  |  |
|  |  | T | 157(34.7) | 236(32.6) |  |  |  |  |  |  |
| HMOX2 | rs2160567 | TT | 88(38.9) | 142(39.3) | 0.992 | 0.901 | 0.373 | 0.301 | 99.16 |  |
|  |  | TC | 107(47.4) | 169(46.8) |  |  |  |  |  |  |
|  |  | CC | 31(13.7) | 50(13.9) |  |  |  |  |  |  |
|  |  | T | 283(62.6) | 453(62.7) | 0.964 |  |  |  |  |  |
|  |  | C | 169(37.4) | 269(37.3) |  |  |  |  |  |  |
| IL-6 | rs1800796 | CC | 99(44.0) | 179(50.1) | 0.323 | 0.843 | 0.310 | 0.282 | 98.31 |  |
|  |  | CG | 101(44.9) | 146(40.9) |  |  |  |  |  |  |
|  |  | GG | 25(11.1) | 32(9.0) |  |  |  |  |  |  |
|  |  | C | 299(66.4) | 504(70.6) | 0.137 |  |  |  |  |  |
|  |  | G | 151(33.6) | 210(29.4) |  |  |  |  |  |  |
| IL-12B | rs3212227 | TT | 49(22.0) | 112(31.3) | 0.029 | 0.410 | 0.466 | 0.432 | 98.14 |  |
|  |  | TG | 119(53.3) | 180(50.3) |  |  |  |  |  |  |
|  |  | GG | 55(24.7) | 66(18.4) |  |  |  |  |  |  |
|  |  | T | 217(48.7) | 404(56.4) | 0.010 |  |  |  |  |  |
|  |  | G | 229(51.3) | 312(43.6) |  |  |  |  |  |  |
| IL-1R1 | rs3732131 | AA | 151(67.1) | 205(56.9) | 0.033 | 0.491 | 0.223 | 0.184 | 98.82 |  |
|  |  | AG | 66(29.3) | 131(36.4) |  |  |  |  |  |  |
|  |  | GG | 8(3.6) | 24(6.7) |  |  |  |  |  |  |
|  |  | A | 368(81.8) | 541(75.1) | 0.008 |  |  |  |  |  |
|  |  | G | 82(18.2) | 179(24.9) |  |  |  |  |  |  |
| IL-10RB | rs3171425 | GG | 105(46.4) | 177(49.3) | 0.657 | 0.523 | 0.302 | 0.306 | 98.82 |  |
|  |  | GA | 103(45.6) | 150(41.8) |  |  |  |  |  |  |
|  |  | AA | 18(8.0) | 32(8.9) |  |  |  |  |  |  |
|  |  | G | 313(69.2) | 504(70.2) | 0.731 |  |  |  |  |  |
|  |  | A | 139(30.8) | 214(29.8) |  |  |  |  |  |  |
| IL-10RA | rs4252249 | GG | 198(87.6) | 327(90.8) | 0.254^a^ | 0.598 | 0.053 | 0.053 | 98.99 |  |
|  |  | GA | 28(12.4) | 32(8.9) |  |  |  |  |  |  |
|  |  | AA | 0(0.0) | 1(0.3) |  |  |  |  |  |  |
|  |  | G | 424(93.8) | 686(95.3) | 0.273 |  |  |  |  |  |
|  |  | A | 28(6.2) | 34(4.7) |  |  |  |  |  |  |
| IL-1RN | rs315952 | CC | 87(38.7) | 117(32.5) | 0.257 | 0.554 | 0.415 | 0.471 | 98.82 |  |
|  |  | CT | 103(45.8) | 174(48.3) |  |  |  |  |  |  |
|  |  | TT | 35(15.5) | 69(19.2) |  |  |  |  |  |  |
|  |  | C | 277(61.6) | 408(56.7) | 0.099 |  |  |  |  |  |
|  |  | T | 173(38.4) | 312(43.3) |  |  |  |  |  |  |
| IL-28B | rs8099917 | TT | 201(89.3) | 333(91.7) | 0.437^a^ | 0.791 | 0.047 | 0.063 | 99.32 |  |
|  |  | TG | 24(10.7) | 29(8.0) |  |  |  |  |  |  |
|  |  | GG | 0(0.0) | 1(0.3) |  |  |  |  |  |  |
|  |  | T | 426(94.7) | 695(95.7) | 0.401 |  |  |  |  |  |
|  |  | G | 24(5.3) | 31(4.3) |  |  |  |  |  |  |
| ^a^ Fisher exact test. SNP, single nucleotide polymorphism; HWE, Hardy-Weinberg equilibrium; MAF, minor allele frequency; OR, odds ratio; CI, confidence interval. | | | | | | | | | | |

| **Supplementary table 10.** Characteristics of three published studies. | | | | | | | | | |
| --- | --- | --- | --- | --- | --- | --- | --- | --- | --- |
| Reference | Region | Test strain | Gene | SNP | Alleles* | Alleles (CHB) | MAF* | MAF(CHB) |  |
| Cummins NW et al. | USA | H1N1 | HMOX1 | rs743811 | T>C | C>T | C=0.353 | T=0.320 |  |
|  |  |  | HMOX2 | rs2160567 | T>C | T>C | C=0.421 | C=0.301 |  |
| Poland GA et al. | USA | H1N1, H3N2, BY | IL-6 | rs1800796 | G>C | C>G | C=0.295 | G=0.282 |  |
|  |  |  | IL-12B | rs3212227 | T>G | T>G | G=0.341 | G=0.432 |  |
|  |  |  | IL-1R1 | rs3732131 | A>G | A>G | G=0.359 | G=0.184 |  |
|  |  |  | IL-10RB | rs3171425 | G>A | G>A | A=0.442 | A=0.306 |  |
|  |  |  | IL-10RA | rs4252249 | G>A | G>A | A=0.186 | A=0.053 |  |
|  |  |  | IL-1RN | rs315952 | T>C | C>T | C=0.215 | T=0.471 |  |
| Egli A et al. | Canada | H1N1, H3N2, BV | IL-28B | rs8099917 | T>G | T>G | G = 0.277 | G=0.063 |  |
| *Data for population of published studies in reference. SNP, single nucleotide polymorphism; MAF, minor allele frequency. | | | | | | | | | |

| **Supplementary table 11.** The minimum sample size in case and control. | | | | | | | | | |
| --- | --- | --- | --- | --- | --- | --- | --- | --- | --- |
| SNPs | Genetic Model | OR | MAF | Controls per case | Population risk | Power | α | Number of cases | Number of controls |
| rs2281929 | Dominant | 0.423 | 0.384 | 365/227=1.61 | (41+193)/1582=0.15 | 0.80 | 0.05 | 71 | 115 |
| rs2455230 | Dominant | 0.535 | 0.476 | 365/227=1.61 | (41+193)/1582=0.15 | 0.80 | 0.05 | 150 | 242 |
| SNP, single nucleotide polymorphism; MAF, minor allele frequency; OR, odds ratio. | | | | | | | | | |

| **Supplementary table 12.** Prediction of splicing variants from SNPinfo. | | | | | | | | |
| --- | --- | --- | --- | --- | --- | --- | --- | --- |
| Gene | SNP | Allele | Position | Prediction Strand | Forward Sequence | Matrix | Score | Method |
| ZBTB46 | rs2281929 | C | 5 | + | GACGCGA | SF2ASF1 | 2.11 | ESEfind |
|  | rs2281929 | C | 5 | + | GACGCGA | SF2ASF1 | 2.11 | ESEfind |
|  | rs2281929 | C | 5 | + | GACGCGA | SF2ASF1 | 2.11 | ESEfind |
|  | rs2281929 | T | 7 | + | GGGACGT | SF2ASF2 | 2.19 | ESEfind |
|  | rs2281929 | T | 7 | + | GGGACGT | SF2ASF1 | 3.96 | ESEfind |
|  | rs2281929 | C | 7 | + | GGGACGC | SF2ASF1 | 2.11 | ESEfind |
|  | rs2281929 | T | 7 | + | GGGACGT | SF2ASF2 | 2.19 | ESEfind |
|  | rs2281929 | T | 7 | + | GGGACGT | SF2ASF1 | 3.96 | ESEfind |
|  | rs2281929 | C | 7 | + | GGGACGC | SF2ASF1 | 2.11 | ESEfind |
|  | rs2281929 | T | 7 | + | GGGACGT | SF2ASF2 | 2.19 | ESEfind |
|  | rs2281929 | T | 7 | + | GGGACGT | SF2ASF1 | 3.96 | ESEfind |
|  | rs2281929 | C | 7 | + | GGGACGC | SF2ASF1 | 2.11 | ESEfind |
| IQGAP2 | rs2455230 | G | 2 | + | TGTATA | SRp55 | 3.61 | ESEfind |
|  | rs2455230 | C | 6 | + | TGCTTCTA | SC35 | 2.95 | ESEfind |
|  | rs2455230 | C | 6 | + | TGCTTC | SRp55 | 3.84 | ESEfind |
